# Changes in clinical presentation, management, and survival outcomes in patients affected by colorectal cancer following COVID-19 pandemic

**DOI:** 10.1093/oncolo/oyae310

**Published:** 2024-11-26

**Authors:** Alessandro Parisi, Riccardo Giampieri, Silvia Villani, Alice Magnarini, Fabio Gelsomino, Donatella Traisci, Francesca Barbin, Lisa Salvatore, Clizia Zichi, Francesca Romana Di Pietro, Federica Zoratto, Andrea Lanese, Angelica Petrillo, Ina Valeria Zurlo, Andrea Spallanzani, Nicola D’Ostilio, Michele Ghidini, Maria Bensi, Francesco Schietroma, Chiara Rognone, Olimpia Panepinto, Jessica Paparo, Teresa Gamba, Renato Bisonni, Sara Di Lorenzo, Bruno Daniele, Giulia Mentrasti, Rossana Berardi

**Affiliations:** Department of Oncology, Università Politecnica delle Marche, Azienda Ospedaliera Universitaria delle Marche, Ancona 60126, Italy; Department of Oncology, Università Politecnica delle Marche, Azienda Ospedaliera Universitaria delle Marche, Ancona 60126, Italy; Department of Oncology, Università Politecnica delle Marche, Azienda Ospedaliera Universitaria delle Marche, Ancona 60126, Italy; Department of Oncology, Università Politecnica delle Marche, Azienda Ospedaliera Universitaria delle Marche, Ancona 60126, Italy; Department of Oncology and Hematology, University Hospital of Modena, Modena 41125, Italy; Medical Oncology, Ospedale San Pio da Pietrelcina, ASL 2 Abruzzo, Vasto 66054, Italy; Oncology Unit, Fondazione IRCCS Ca’ Granda Ospedale Maggiore Policlinico, Milan 20122, Italy; Comprehensive Cancer Center, Fondazione Policlinico Universitario “A Gemelli”—IRCCS, Rome 00168, Italy; Medical Oncology, Catholic University of Sacred Heart, Rome, Italy; Department of Oncology, University of Turin, Ordine Mauriziano Hospital, Turin 10128, Italy; U.O.C. Oncologia, Istituto Dermopatico dell’Immacolata IDI-IRCCS 00167, Roma, Italy; Medical Oncology Unit, Ospedale “S.M. Goretti”—ASL Latina 04100, Latina, Italy; UOC Oncologia Medica, Ospedale A. Murri 63900, Fermo, Italy; Medical Oncology Unit, Ospedale del Mare, Naples, Italy; Medical Oncology, “Vito Fazzi” Hospital, Lecce 80147, Italy; Department of Oncology and Hematology, University Hospital of Modena, Modena 41125, Italy; Medical Oncology, Ospedale San Pio da Pietrelcina, ASL 2 Abruzzo, Vasto 66054, Italy; Oncology Unit, Fondazione IRCCS Ca’ Granda Ospedale Maggiore Policlinico, Milan 20122, Italy; Medical Oncology, Catholic University of Sacred Heart, Rome, Italy; Medical Oncology, Catholic University of Sacred Heart, Rome, Italy; Department of Oncology, University of Turin, Ordine Mauriziano Hospital, Turin 10128, Italy; Department of Oncology, University of Turin, Ordine Mauriziano Hospital, Turin 10128, Italy; Department of Oncology, University of Turin, Ordine Mauriziano Hospital, Turin 10128, Italy; Department of Oncology, University of Turin, Ordine Mauriziano Hospital, Turin 10128, Italy; UOC Oncologia Medica, Ospedale A. Murri 63900, Fermo, Italy; Medical Oncology Unit, Ospedale del Mare, Naples, Italy; Medical Oncology Unit, Ospedale del Mare, Naples, Italy; Department of Oncology, Università Politecnica delle Marche, Azienda Ospedaliera Universitaria delle Marche, Ancona 60126, Italy; Department of Oncology, Università Politecnica delle Marche, Azienda Ospedaliera Universitaria delle Marche, Ancona 60126, Italy

**Keywords:** colorectal cancer, COVID-19, COVID-DELAY, diagnostic delay, therapeutic delay, survival, multidisciplinary discussion, staging

## Abstract

**Background:**

As an extended analysis of the COVID-DELAY study, we aimed to assess the impact of the COVID-19 pandemic on diagnosis, staging, and survival outcomes among patients with colorectal cancer (CRC) diagnosis performed from 2019 to 2022.

**Methods:**

All consecutive newly diagnosed CRC patients referred to 11 Italian Oncology Departments between March and December 2019, 2020, 2021, and 2022 were enrolled. Access rate, demographics, diagnostic-therapeutic temporal intervals, and first-line progression-free survival (PFS) and OS among metastatic patients were assessed.

**Results:**

Compared to 2019 (*n* = 690), an initial global reduction in new CRC cases in 2020 (*n* = 564, –18.3%) was observed, followed by a progressive increase in new CRC diagnoses in 2021 (*n* = 748, + 8.4%) and 2022 (*n* = 756, + 9.6%); a higher rate of TNM stage IV tumors was diagnosed in 2020 (35.4%) and 2021 (31.0%) compared to 2019 (29.6%), with normalization in 2022 (26.4%) (*P* < .001). Not clinically relevant differences between histological diagnosis and first oncological examination, cytohistological diagnosis and systemic treatment start, first oncological appointment and systemic treatment start, treatment start and first radiological assessment between 2020 and 2021-2022 years were found. After propensity score matching according to the year of diagnosis, median OS was significantly worse in 2020, 2021, and 2022 compared to 2019 (27.6 vs 24.8 vs not reached vs 38.9 months, respectively) (*P* < .001). Concordantly, the median PFS was significantly worse with each passing year: 13.0 vs 11.1 vs 9.2 vs 7.2 months in 2019, 2020, 2021, and 2022, respectively (*P* = .00027).

**Conclusions:**

A progressive normalization in the rate of new CRC diagnosis as well as TNM stages at diagnosis, in 2021 and 2022 compared to 2020 and 2019, was found. The increase in new CRC cases might have affected some diagnostic-therapeutic time intervals in 2021-2022 years compared to 2020. Significantly, compared to the pre-pandemic phase, pandemic years were independently associated with worse PFS and OS outcomes in patients affected by metastatic disease.

**Implications for practice**

This study retrospectively involved several Italian cancer centers managing patients affected by colorectal cancer (CRC) across the pandemic years from 2019 to 2022. After an initial drop, the authors found a progressive normalization in the rate of new CRC diagnosis as well as TNM stages at diagnosis, in 2021 and 2022 compared to 2020 and 2019. The increase in new CRC cases might have affected some diagnostic-therapeutic time intervals in 2021-2022 years compared to 2020. Significantly, in patients affected by metastatic disease, pandemic years were independently associated with worse PFS and OS outcomes compared to the pre-pandemic phase. These observations further identify among others, metastatic CRC patients as a frail population at higher risk of death from any cause during such a severe pandemic event, and can help in properly defining public health strategies for the future.

## Introduction

From the first rumblings in Hubei province to its brakeless worldwide spread, coronavirus disease 2019 (COVID-19) has represented one of the worst pandemics of the modern era.

Italy was the first Western Country to face the COVID-19 outbreak, experiencing a severe increase in terms of new cases and deaths, particularly during the first pandemic wave. Mitigation efforts such as lockdowns of institutions until a complete reorganization of the National Health System, including reallocation of crucial human and economic health resources toward COVID-19 patient care pathways, were carried out to limit pandemic incidence and mortality and to face this unprecedented scenario.^1,2^ This inevitably impacted hospital admissions for non-communicable diseases, hampering both inpatients and outpatients care.

As a consequence, many diagnostic and therapeutic services in non-COVID-19-related care activities such as cancer screening and surgery have been deferred or canceled.^3,4^

According to national statistics, colorectal cancer (CRC) stands as the second leading cause of cancer death regardless of gender.^5^ However, the large-scale adoption of screening programs and the implementation in the clinical practice of multidisciplinary diagnostic-therapeutic pathways have significantly impacted CRC prognosis.^6^

In a preliminary experience of the COVID-DELAY study, a decline in CRC diagnoses in 2020, together with the rising incidence of CRC at an advanced stage compared to 2019, was found. On the other hand, Italian Oncology Departments guaranteed the tightness of diagnostic-therapeutic pathways and access to care in CRC patients, mitigating the effects of COVID-19.^7^

Patients with cancer appeared at increased risk of contracting SARS-CoV2 infection and developing more severe disease course and sequelae alongside an increased risk of death.^8–11^ The risk of higher tumor burden in patients affected by metastatic CRC (mCRC), together with the above-mentioned increased risk of death or sequelae, might have limited the systemic treatment effectiveness in terms of survival outcomes during the COVID-19 pandemic years.

SARS-CoV-2 universal vaccination and boosting of immunity, together with the enhancement of public health measures and improvement management of the disease, led to a significant improvement in COVID-19-related outcomes particularly in patients affected by hematological and solid malignancies.^12,13^

Poor data concerning the effect of the COVID-19 pandemic on diagnostic-therapeutic pathways and survival outcomes during the vaccination and the post-emergency pandemic phase are available to date. The present analysis aimed to assess the effects of COVID-19 impact on the diagnosis, staging, and treatment outcomes of CRC patients diagnosed and managed in different Italian regions across the pandemic and post-pandemic years.

## Methods

### Study design and population

All consecutive newly diagnosed CRC patients referred to 11 Italian Oncology Departments between March and December 2019 (pre-pandemic phase), 2020 (acute pandemic phase), 2021 (vaccination phase), and 2022 (post-emergency pandemic phase) were evaluated within the COVID-DELAY study (“Evaluation of COVID-19 impact on DELAYing diagnostic-therapeutic pathways of cancer patients in Italy”).^7^ The Oncology Departments were located across Northern (3 academic hospitals), Central (3 academic and 3 secondary care hospitals), and Southern (2 secondary care hospitals) urban regions of Italy. The present analysis aimed to estimate the difference in terms of diagnosis and treatment from 2019 to 2022, by assessing the total number of new diagnoses per year, and temporal intervals between the date of symptoms onset, radiological and cytohistological diagnosis, first oncological appointment, treatment start, and first radiological reassessment. Differences in patients and disease characteristics as well as in progression-free survival (PFS) and overall survival (OS) among patients affected by mCRC were also assessed.

Ethical approval to conduct this study was obtained by the local ethical committees on human experimentation of each participating center, after previous approval by the coordinating center (“Comitato Etico Regionale delle Marche—C.E.R.M.,” Reference Number 2021 139). The present study complies with the provisions of the Good Clinical Practice guidelines and the Declaration of Helsinki and local laws and fulfills Regulation (EU) 2016/679 of the European Parliament and the Council of April 27, 2016, on the protection of natural persons concerning the processing of personal data.

Patients were included if they were 18 years or older, had histologically proven diagnosis of CRC performed between March and December 2019, 2020, 2021, and 2022, and received at least one type of oncological treatment (either surgery, radiotherapy, or systemic therapy) after diagnosis, and had available data about radiological diagnosis, cytohistological diagnosis, and treatment start. Patients with recurrent CRC or gastrointestinal (GI) malignancies other than CRC were excluded.

Temporal intervals between the date of symptoms onset, radiological diagnosis, cytohistological diagnosis, first oncological appointment, treatment start, and first radiological reassessment of each patient with CRC diagnosis performed from March to December 2019, 2020, 2021, 2022 were computed and compared with each other. To avoid negative values, data of patients who had their CRC diagnosis after the first oncological appointment (as per the standard practice of referral Hospitals) were not included in the calculation of these specific temporal intervals. Baseline (at diagnosis) data about the patient, tumor, and treatment characteristics were also retrieved from medical records and differences were analyzed.

Subgroup analyses were performed by investigating the study aims according to the regions (Northern, Central, and Southern Italy) of the Oncology Department where patients with CRC were managed.

### Statistical analysis

Descriptive statistics were used to report baseline patient, disease, and treatment characteristics. Differences between categorical variables were analyzed by exact Fisher test or chi-square, as appropriate, while differences between continuous variables were evaluated by Student’s *t*-test or Mann–Whitney U test as applicable.

PFS was calculated starting from the first cycle of chemotherapy until the last contact for patients alive, the patient’s death or first sign of disease progression, or the last visit for lost-to-follow-up patients. OS was calculated starting from the first cycle of chemotherapy until the patient’s death or the last visit for lost-to-follow-up patients. Survival was calculated by the Kaplan–Meier method, and association with variables was assessed by log-rank test. Multivariate analysis was performed by Cox regression.

Multivariate analysis was performed by taking into account stratification factors that were described previously: sex, age with 2 different cutoffs (early onset: <50 years old, standard onset: 50–75 years old, elderly: >75 years old), regions of Italy (Northern vs Central vs Southern), primary tumor sidedness (right vs left vs rectum), KRAS/NRAS/BRAF mutation (all wild-type vs BRAF mutant vs RAS mutant), high microsatellite instable (MSI-H)/deficient mismatch repair proteins (dMMR) status (yes vs not), Eastern Cooperative Oncology Group performance status (ECOG-PS) at treatment start (0 vs 1 vs 2–3), and whether the diagnosis was performed during emergency ward admittance (yes vs not).

Propensity score matching was performed by taking into account all the above-mentioned stratification factors after being dichotomized (year of diagnosis 2019: yes vs not, sex: male vs female, Italian region: Center vs not, BRAF mutation: yes vs not, RAS mutation: yes vs not, right-side tumor: yes vs not, ECOG-PS 0: yes vs not, elderly age: yes vs not, early onset CRC: yes vs not, emergency ward admission at diagnosis: yes vs not, MSI-H/dMMR status: yes vs not). The method used was “nearest,” the caliper was set at 2, and the ratio was set at 1.

The alpha level for all analyses was set to *P* < .05.

For all calculations, we used IBM SPSS Statistics, version 26.0 (released 2019, IBM SPSS Statistics for Macintosh, version 26.0; IBM Corp.), MedCalc® Statistical Software version 19.7.2 (MedCalc Software Ltd; https://www.medcalc.org; 2021), and R statistical software (version 4.1.2) (with loaded packages MatchIt, survival, survminer, logistf).

## Results

### Population

**Figure 1.**
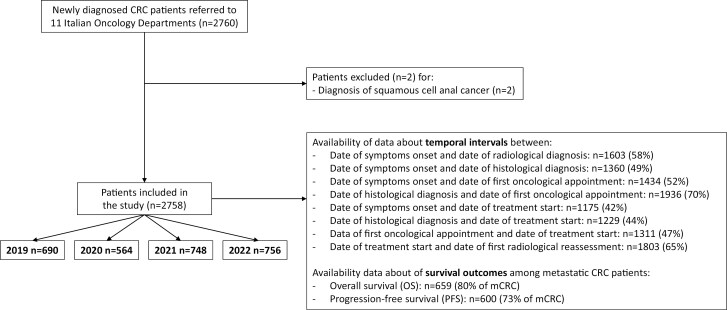
CONSORT diagram with patient’s selection and disposition according to the availability of data concerning diagnostic-therapeutic temporal intervals and survival outcomes.

A total of 2758 patients affected by CRC at any stage were included in the present analysis (Figure 1).

Compared to 2019 (*n* = 690), a reduction in new CRC cases was found in 2020 (*n* = 564, −18.3%). Conversely, a progressive increase in new CRC diagnoses was found in 2021 (*n* = 748, +32.6%) and 2022 (*n* = 756, +34.0%), compared to 2020.

**Figure 2.**
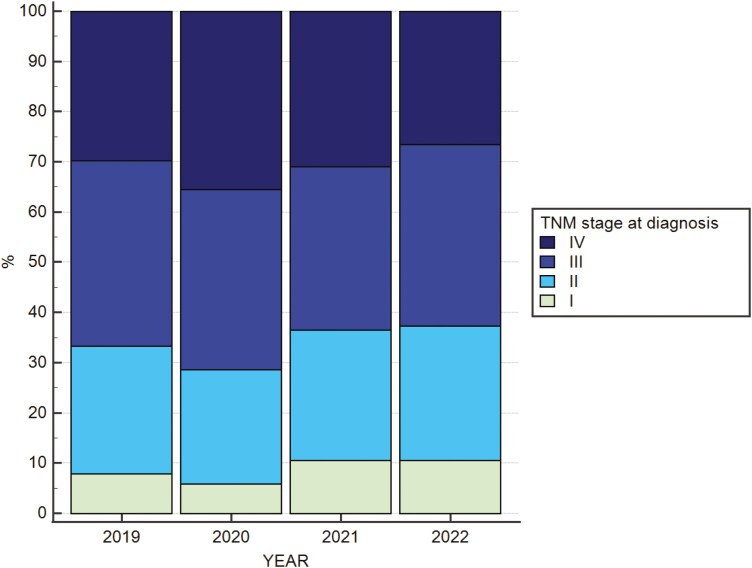
TNM stage according to the year of diagnosis.

Regarding tumor and patients’ characteristics, compared to 2019 (29.6%), a higher rate of TNM stage IV tumors was diagnosed in 2020 (35.4%) and 2021 (31.0%), with normalization in 2022 (26.4%) (*P* < .001) (Figure 2).

Focusing on the TNM stage at diagnosis according to the different regions of Italian oncology departments, a statistically significant difference, regardless of year of diagnosis, was found. Particularly, compared to the Central and Southern regions, a higher rate of earlier CRC diagnoses was found in Northern Italy. Indeed, TNM stage I cases were 144/1004 (14%), 78/1136 (7%), and 20/564 (3%), meanwhile TNM stage IV cases were 271/1004 (27%), 312/1136 (27%), and 237/564 (42%) in the Northern, Central, and Southern Italy, respectively (*P* < .0001) (Figure S1).

When regions were assessed separately, differences in the TNM stage at diagnosis were also evident.

In Northern Italy, TNM stage IV diagnoses were 47/236 (20%), 73/169 (43%), 89/291 (30%), and 62/308 (20%) in 2019, 2020, 2021, and 2022 respectively (*P* < .0001) (Figure S2).

On the other hand, no statistically significant differences in TNM stage at diagnosis were found in Central Italy: TNM stage IV cases were 83/316 (26 74/%), 271 (27%), 80/266 (30%), 75/283 (75%) in 2019, 2020, 2021, and 2022, respectively (*P* = .08).

Finally, a statistically significant change in TNM stage at diagnosis was found in Southern Italy: TNM stage IV diagnoses were 72/128 (56%), 51/119 (43%), 57/173 (33%), and 61/157 (39%) in 2019, 2020, 2021, and 2022, respectively (*P* = .0041) (Figure S3).

Intriguingly, a higher rate of patients performed oncological diagnosis after access to the emergency room in 2021 (32.3%) compared to 2019 (25.0%) and 2020 (27.2%), with normalization in 2022 (26.3%) (*P* = .023). Overall, a lower number of patients has been discussed in multidisciplinary tumor boards (MTBs) in 2020 (35.6%) compared to 2019 (45.4%), 2021 (47.5%), and 2022 (55.0%) (*P* < .001). Intriguingly, a higher rate of mucinous tumors was diagnosed in 2021-2022 (12.5%–12.3%) compared to 2019-2020 (5.3%–5.2%) (*P* < .001), with a similar higher rate of dMMR/MSI-H in 2021-2022 compared to previous years (9.5%–15.5% vs 7.6%–7.9%, *P* < .001). According to region, during 2021-2022 compared to 2019-2020 years, a lower rate of new cancer diagnoses was performed at the Oncology departments of Northern Italy compared to those of Central Italy (*P* < .001) (Table 1). Among all the patients included, 40 were not Caucasian (namely, 13 African-American patients and 27 Asian patients). No differences in gender, age (including the incidence of early-onset CRC compared to average-onset CRC), ECOG-PS, sidedness, *RAS/BRAF* mutational status, treatment with radiotherapy, and inclusion in clinical trials across years were found.

**Table 1.** Patients’ characteristics according to the year of diagnosis.

Characteristics	2019 *N* (%)	2020 *N* (%)	2021 *N* (%)	2022 *N* (%)	*P-*value
**Patients**	690	564	748	756	
**Sex**					
* Female*	290 (42.0)	262 (46.5)	348 (46.5)	349 (46.2)	.266
* Male*	400 (58.0)	302 (53.5)	400 (53.5)	407 (53.8)	
**Age, years, median (range)**	70 (28–95)	69 (21–92)	70 (26–94)	70 (27–96)	-
**Onset of CRC**					
* EO-CRC* (*< 50 years old*)	649 (94.1)	532 (94.3)	712 (95.2)	717 (94.8)	.784
* AO-CRC* (*≥ 50 years old*)	41 (5.9)	32 (5.7)	36 (4.8)	39 (5.2)	
**ECOG-PS at the start of treatment**					
* 0*	298 (61.8)	269 (63.4)	356 (61.2)	340 (56.1)	.353
* 1*	156 (32.4)	131 (30.9)	190 (32.6)	216 (35.6)	
* 2*	23 (4.8)	20 (4.7)	26 (4.5)	42 (6.9)	
* 3*	5 (1.0)	4 (0.9)	10 (1.7)	8 (1.3)	
**Sidedness**					
* Right*	286 (42.8)	225 (40.2)	302 (40.5)	314 (41.6)	.602
* Left*	297 (44.4)	275 (49.1)	353 (47.3)	340 (45.0)	
* Rectum*	86 (12.9)	60 (10.7)	91 (12.2)	101 (13.4)	
**Tumor histology**					
* Adenocarcinoma*	646 (94.3)	527 (94.3)	645 (86.8)	659 (87.2)	<.001
* Mucinous*	36 (5.3)	29 (5.2)	93 (12.5)	93 (12.3)	
* Neuroendocrine cancer* (*NEC*)	3 (0.4)	3 (0.5)	5 (0.7)	4 (0.4)	
**Stage at diagnosis**					
* I*	56 (8.2)	33 (5.9)	77 (10.5)	80 (10.7)	<.001
* II*	173 (25.4)	127 (22.7)	190 (26.0)	200 (26.7)	
* III*	251 (36.8)	201 (36.0)	237 (32.5)	271 (36.2)	
* IV*	202 (29.6)	198 (35.4)	226 (31.0)	198 (26.4)	
**Diagnosis performed after access to first aid**					
* Yes*	116 (25.0)	99 (27.2)	227 (32.3)	188 (26.3)	.023
* No*	348 (75.0)	265 (72.8)	476 (67.7)	526 (73.7)	
**Mutational status**					
* RAS/BRAF wild-type*	169 (54.3)	142 (49.8)	165 (47.6)	162 (50.2)	.161
* RAS mutant*	116 (37.3)	119 (41.8)	149 (42.9)	118 (36.5)	
* BRAF mutant*	26 (8.4)	24 (8.4)	33 (9.5)	43 (13.3)	
**MMR/MSI status**					
* pMMR/MSS*	305 (92.4)	326 (92.1)	534 (90.5)	491 (84.5)	<.001
* dMMR/MSI-H*	25 (7.6)	28 (7.9)	56 (9.5)	90 (15.5)	
**Treatment setting**					
* Neoadjuvant* (*including CTRT*)	85 (12.5)	83 (15.0)	100 (14.9)	101 (14.0)	<.001
* Adjuvant*	182 (26.7)	169 (30.6)	202 (30.2)	247 (34.2)	
* Metastatic*	168 (24.6)	160 (28.9)	190 (28.4)	176 (24.4)	
* Adjuvant post-metastasectomy* (*NED*)	5 (0.7)	11 (2.0)	13 (1.9)	8 (1.1)	
* Follow-up*	241 (35.3)	128 (23.1)	161 (24.1)	190 (26.3)	
* Best supportive care*	1 (0.1)	2 (0.4)	3 (0.4)	0 (0.0)	
**Radiotherapy**					
* Yes*	77 (13.4)	75 (15.9)	96 (13.2)	96 (12.9)	.475
* No*	499 (86.6)	397 (84.1)	631 (86.8)	647 (87.1)	
**MTD discussion**					
* Yes*	313 (45.4)	198 (35.6)	350 (47.5)	410 (55.0)	<.001
* No*	376 (54.6)	358 (64.4)	387 (52.5)	335 (45.0)	
**Inclusion in clinical trials**					
* Yes*	26 (4.1)	14 (2.7)	24 (3.2)	21 (2.8)	.455
* No*	605 (95.9)	511 (97.3)	719 (96.8)	732 (97.2)	
**Region according to Department site**					
* North*	319 (46.2)	272 (48.2)	271 (36.2)	289 (38.2)	<.001
* Center*	241 (34.9)	171 (30.3)	304 (40.6)	311 (41.1)	
* South*	130 (18.8)	121 (21.5)	173 (23.1)	156 (20.6)	

*P*-values were calculated excluding the unknown values.

Abbreviations: AO-CRC, average-onset colorectal cancer; CTRT, concurrent chemo-radiation therapy; dMMR/MSI-H, mismatch repair deficient/microsatellite instability high; ECOG-PS, Eastern Cooperative Oncology Group performance status; EO-CRC, early-onset colorectal cancer; MMR/MSI, mismatch repair/microsatellite instability; MTD, multidisciplinary team; NED, not evidence of disease; pMMR/MSS, mismatch repair proficient/microsatellite stable.

### Diagnostic-therapeutic time intervals

Looking at patients’ management, significant differences in terms of the temporal interval between histological diagnosis and the first oncological examination, histological diagnosis and systemic treatment start, and the first oncological appointment and systemic treatment start across the 4 years were found (Table 2). This variation was mostly led by a significant difference between histological diagnosis and the first oncological examination (median of 30 vs. 38 days, respectively, *P* < .001), cytohistological diagnosis and systemic treatment start (median of 49 vs. 58 days, *P* < .001), the first oncological appointment and systemic treatment start (median of 14 vs. 16 days, *P* = .007), treatment start and the first radiological assessment (median 96 vs. 105 days, *P* = .027) between 2020 and 2021-2022 cohort, respectively (Table S1).

**Table 2.** Temporal intervals between the date of symptoms onset, radiological diagnosis, cytohistological diagnosis, first oncological appointment, treatment start, and first radiological reassessment between 2019, 2020, 2021, and 2022.

Time interval	2019 Median, days (IQR)	2020 Median, days (IQR)	2021 Median, days (IQR)	2022 Median, days (IQR)	*P*-value^a^
Symptom onset/radiological diagnosis	25 (54)	20 (58)	21 (46)	25 (44)	.028
Symptom onset/cytohistological diagnosis	31 (47)	28 (62)	27.5 (49)	25 (46)	.042
Symptom onset/first oncological appointment	79 (63)	69 (65)	76 (62)	72 (63)	.126
Cytohistological diagnosis/first oncological appointment	39 (37)	30 (30)	38 (32)	39 (36)	<.001
Symptom onset/treatment start	99 (86)	91 (78)	98 (77)	90 (67)	.057
Cytohistological diagnosis/treatment start	59 (42)	49 (43)	57 (44)	58 (34)	<.001
First oncological appointment/treatment start	17 (20)	14 (16)	19 (19)	16 (15)	.042
Treatment start/first radiological assessment	105 (97)	96 (87)	106 (96)	104 (56)	.181

^a^Kruskal–Wallis H test comparing time intervals among them in 2019, 2020, 2021, and 2022 years. *P*-values were calculated excluding patients with unknown values. Statistically significant (*P* < .05).

Abbreviation: IQR, interquartile range.

### Survival analysis

A total of 659 stage IV patients were evaluable for OS analysis, and 600 patients were evaluable for first-line PFS analysis. At a median follow-up time of 21.2 (95% CI, 19.5−22.8) months in the overall population, 432/600 (72%) patients progressed after first-line treatment, and 261/659 (40%) patients have already died. Median follow-up time was 41.2 (95% CI, 39.0−43.4), 29.7 (95% CI, 28.4−31.1), 18.9 (95% CI, 17.9−19.8), and 7.0 (95% CI, 6.1−7.9) months in 2019, 2020, 2021, and 2022 cohorts, respectively.

In the overall population, the first-line median OS (mOS) was 26.74 (95% CI, 24.4−30.8) months, while the first-line median PFS (mPFS) was 9.77 (95% CI, 9.2−10.5) months.

**Figure 3.**
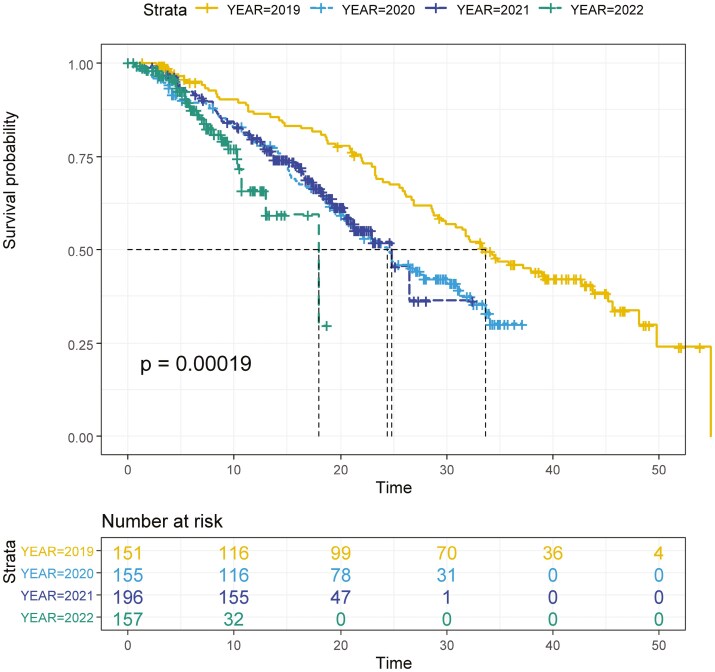
Kaplan–Meir curves for OS according to the year of diagnosis.

After stratification according to the year of diagnosis, a statistically significant difference in mOS between mCRC patients diagnosed in 2019 (33.6 months, 95% CI, 29.2−42.7), 2020 (24.4 months, 95% CI, 20.3−30.0 months), 2021 (24.8 months, 20.5−26.5 months), and 2022 (18.0 months, 95% CI, 12.9−18.0) (*P* = .0019) was found (Figure 3).

**Figure 4.**
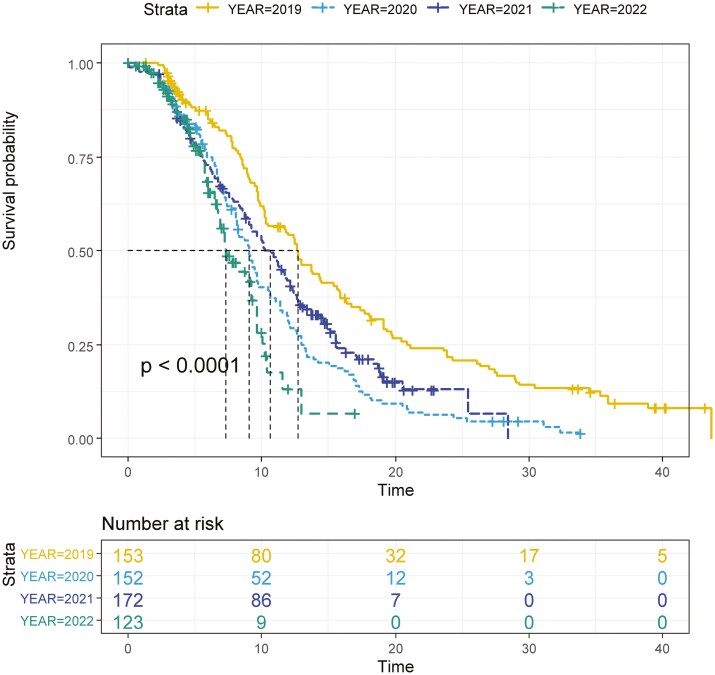
Kaplan–Meir curves for PFS according to the year of diagnosis.

Similarly, a statistically significant difference in mPFS between mCRC patients diagnosed in 2019 (12.7 months, 95% CI, 10.2−14.4), 2020 (9.1 months, 95% CI, 8.1−9.7), 2021 (10.6 months, 9.0−11.8 months), and 2022 (7.3, 6.7−9.2 months) (*P* < .0001) was found (Figure 4).

Multivariate analysis for OS confirmed an independent negative prognostic role of the year of diagnosis (worse OS in 2020, 2021, and 2022, each compared to 2019) with an incremental negative prognostic impact with each passing year. Intriguingly, an independent prognostic role of the Italian region was found, while the prognostic role of ECOG-PS status and RAS mutations was confirmed (Table S2).

Concordantly, multivariate analysis for PFS confirmed the independent prognostic role of the year of diagnosis, with worse PFS in 2020, 2021, and 2022, each compared to 2019. As expected, an independent prognostic role was confirmed for ECOG-PS as well as for BRAF and KRAS mutations. Intriguingly, an independent prognostic role according to Italian region categorization was found again. Early-onset CRC patients (< 50 years old) seemed to have better PFS compared to standard onset patients (Table S2).

After propensity score matching according to the year of diagnosis (2019 vs 2020-2021-2022), out of 423 control units to be matched with 92 units of the 2019 cohort, 331 were discarded (Jitter plot and Histogram plot are shown in Figure S4A and B).

When survival analysis was performed in the matched cohort, mOS was still significantly worse in 2020, 2021, and 2022 compared to 2019 (27.6 [95% CI,15.36−27.83] vs 24.8 [95% CI, 17.50−24.83] vs not reached (NA) vs 38.9 [95% CI, 32.20−48.09] months, respectively) (*P* < .001) (Figure S5).

Concordantly, PFS was also significantly worse with each passing year: 13.0 (95% CI, 10.33−16.25) vs 11.1 (95% CI, 7.70−12.99) vs 9.2 (95% CI, 7.14−11.25) vs 7.2 (95% CI, 6.02−10.03) months in 2019, 2020, 2021, and 2022, respectively (*P* = .00027) (Figure S6).

## Discussion

The COVID-19 outbreak has unprecedentedly changed the face of cancer care and permanently shaped the global healthcare landscape.

With our country at the forefront of such an unparalleled struggle, Italian oncologists were expected to lead their patients through the eye of the storm, weighing the risks and benefits of giving cancer treatment compared to the chance of getting them infected with COVID-19.^14^

Furthermore, patients with cancer had to fight a struggle on multiple fronts: on one hand, facing the fear of contracting COVID-19, with the risk of developing potentially severe or fatal complications, particularly in defined clinical settings^9–11^; on the other hand, dealing with the uncertainty of deferred elective oncological procedures as well as treatment plan discontinuations or adaptations.

Particularly in 2020, with a healthcare system close to collapse and limited experience-based guidelines and recommendations, medical oncologists’ associations had to elaborate a prompt response. In this respect, contrasting measures have been adopted to effectively manage the crisis, such as patient-tailored reconsideration of treatment indication and schedule adaptation to reduce avoidable hospital admission, visits’ conversion to telehealth encounters, and multidisciplinary board rearrangements following reallocation to COVID-19 units.^15^

Under this point of view, despite the earliest establishment of experts’ consensus and the implementation of these recommendations in daily clinical practice, the outcome of the efforts made to prevent diagnostic delays and the much-feared “upstaging effect” was a matter of speculation and might have affected the subsequent years.^15,16^

Our analysis was thereafter intended to explore the effects on the expected cancer incidence as well as on cancer diagnostic-therapeutic pathways and survival rates, these diversions may have led to, during the post-pandemic phase.

In the first part of our analysis, a worsening drop in CRC diagnoses in 2020 compared to 2019 was confirmed.^7^ This trend was in line with that reported by most of the 43 studies included in a recent systematic review investigating the effect of the COVID-19 pandemic on the diagnosis and treatment of CRC.^17^ Many factors might have contributed to this reduced number of diagnoses: lockdowns and fear of contagion might have deterred people symptomatic for CRC to ask for help and ultimately to undergo colonoscopy and instrumental assessment to properly diagnose and stage this disease. This fact might have led to late CRC diagnoses for patients who were symptomatic and thus a higher risk of a larger tumor burden and more advanced disease at the time of diagnosis. Furthermore, this would justify the higher rate of new diagnoses after first aid access in 2021, a result that is consistent with previous findings.^17^ On the other hand, as screening programs were suspended during the pandemic, CRC screening performed by fecal immunochemical test followed by colonoscopy was also temporarily halted: this might have led to a reduction in the number of early CRC diagnoses, mainly in the group of patients who were asymptomatic for this disease.

Within the present updated analysis, we highlight a worrying drop in terms of new CRC diagnoses during the vaccination phase and the post-emergency pandemic phase. Most strikingly, a significantly higher incidence of late-stage compared to early-stage CRC diagnoses in 2021 as well as in 2020 compared to 2019 was found, with a trend toward normalization in the post-emergency pandemic phase. It is easy to hypothesize that this would be the number of patients who were so symptomatic they could not avoid asking for help and who would be diagnosed with metastatic disease involvement. Indeed, if we look at what happened in the following years (2021-2022), the number of CRC diagnoses increased more due to a higher number of patients with early-stage disease, rather than a net increase in the number of patients with metastatic disease. Taken together, these findings are consistent with incidence data at a national level (49 000 new cases in 2019 vs 43 700 new cases in 2020 vs 48 100 new cases in 2022—updated and realistic data on incidence not available for the 2021 year because of the consequences led by the COVID-19 pandemic) and would indicate a gradual return to normality after a setback in the Italian healthcare system for screening and diagnostic ability, alongside a certain reluctance of many patients to seek healthcare in crowded healthcare centers, during both acute pandemic and vaccination phase.^5,18^ Interestingly, this could also explain the marked differences in stage at diagnosis that could be observed by comparing different geographical areas of Italy. If we assume that CRC screening should be considered an effective tool for early diagnosis of CRC, we would have expected that the suspension of screening programs would have the greatest impact on those areas where compliance with screening was the highest. Indeed, data from the Italian “Osservatorio Nazionale Screening” showed that adherence to CRC screening was around 40.5% in 2019, compared to 34.1% in 2020 and 38.7% in 2021.^19^ However, marked differences between Italian regions were found. Indeed, from 2019 to 2020, compliance with screening programs in Northern, Central, and Southern Italy decreased from 49.4% to 46.8%, from 34.8% to 27.2%, and from 25.7% to 15.8%. Since the detection rate for cancer usually ranges around 0.08%−0.2%, it can be expected that the decrease in early diagnoses would be more marked in Northern Italy compared to Central and Southern Italy, as our data seem to suggest.

Despite the hard times, our results further proved that Italian medical oncologists met the challenge of preventing cancer patients from being left orphans of care. Indeed, no particular leakage in the management system of CRC patients emerged in terms of temporal intervals of the diagnostic-therapeutic pathway. Paradoxically, a reduced time was found in terms of some temporal intervals during 2020 compared to 2019 (ie, between cytohistological diagnosis and first oncological examination, first oncological appointment, and systemic treatment start) and, sometimes, compared to 2021 and 2022 years. At least in part, this might be related to the reduced number of new cancer patients diagnosed in 2020, easing the pressure on a pandemic-distressed system and accelerating patients’ encounters compared to 2019 as well as to 2021 and 2022 years. Additionally, the late-stage presentation shown after COVID-19, generally precluding a surgical approach, might have hastened the referral to medical oncologists. Moreover, this unexpected and positive trend in 2020 patient management might have also been related to the extensive use of telemedicine and supported by the firm resilience of healthcare providers, as demonstrated by multiple resources.^14,20,21^ On the other hand, the consistent increase in CRC cases might have affected some diagnostic-therapeutic time intervals during the vaccination and post-emergency pandemic phases compared to the pandemic phase.

With the MTB approach representing the best practice in the management and decision-making for cancer patients worldwide,^22^ COVID-19 pandemic limitations have imposed technical, financial, and relational issues.^23–25^ Intriguingly, after an initial setback of multidisciplinary discussion of CRC patients with a significant decrease in the rate of the cases reviewed in 2020 compared to 2019, the activity of MTBs progressively improved, even exceeding the pre-pandemic numbers. Of course, the introduction of properly regulated videoconferences as an alternative form of communication among medical professionals in routine MTB, while reducing the need for traveling time to conference rooms, might have helped to preserve and increase the rate of CRC patient cases properly shared and discussed.

The COVID-19 pandemic triggered a brisk contraction of clinical research in Italy and globally.^26,27^ This drop in patient recruitment has been related to the decreased ability of clinical, support, and preclinical units to provide nonessential activities and to the reallocation of resources to more critical services and trials.^28^ With regard to the centers involved in the present study, no statistically significant difference was found in terms of the rate of patients enrolled in clinical trials across years from the pre-pandemic and the post-emergency pandemic phases.

One of the most interesting findings of our analysis is that concerning the alarming worsening of the prognosis of patients with stage IV CRC during the SARS-Cov2 pandemic years: despite the introduction of novel treatment modalities for patients with stage IV CRC in the last years (ie, rechallenge or reintroduction with anti-EGFR for liquid biopsy-proven RAS wild-type patients,^29^ encorafenib plus cetuximab treatment for BRAF V600E mutated patients,^30,31^ and immune checkpoint inhibitors for patients with MSI-H/dMMR mCRC^32,33^), both first-line PFS and OS were increasingly worse with each passing year. This negative prognostic effect was confirmed after multivariate analysis and matching for all those stratification factors that are usually considered to have an impact on both PFS and OS (tumor sidedness, RAS and BRAF mutational status, ECOG-PS at treatment start).

There might be a few explanations for this. At least in part, the higher risk of disease progression and death during the pandemic years might be related to changes in treatment plans, including changes to less effective systemic regimens to limit the risk of particularly hematological, treatment-related adverse events, as well as to the lower dose intensity of anticancer drugs, which the pandemic phase might have led to (ie, treatment discontinuation because of COVID-19 infection, limited access to day hospitals, reducing of day hospital “seats” for those patients undergoing to palliative chemotherapy, and so on).^15,17,34,35^ Indeed, we observed that the same treatment modalities that were used in the first-line setting did yield significantly worse outcomes after 2019, thus suggesting that something related to how the treatment was performed might be responsible for the reduced effect.

Even though the negative impact on oncology wards and inpatient clinic activity was massive, the reduction in activity of surgical wards was even more marked. CRC prognosis is highly dictated also by radicality and quality of surgery, as previous studies have suggested. Indeed, primary tumor resection even in the metastatic disease setting and surgical management of the oligometastatic disease are stapled measures that have contributed to increasing OS of patients with mCRC. Indeed, although the mOS of patients who receive the best medical treatment options nowadays is estimated to be around 30–33 months, it easily ranges around 60–70 months for patients who undergo surgical resection of the primary tumor and metastases.^36,37^ Despite that, we could not prove this: the proportion of patients with stage IV diagnosis that underwent surgical resection of the primary tumor was 60% in 2019 vs 67% in 2020 vs 82% in 2021 vs 76% in 2022. This would seemingly suggest that, despite all limitations to surgery in the pandemic years, the number of metastatic patients who were able to receive primary surgery was not reduced with each passing year. Resection of metastatic sites in this unselected population was 2%–3% and was maintained the same throughout the years.

Another factor that might explain this reduced life expectancy is the one linked to greater tumor burden at diagnosis, as later diagnosis might mean an increased size of the tumor at the time of discovery. There is no official consensus concerning how to reliably and reproducibly assess the size of tumor involvement and this might partly explain why this information is usually lacking in most analyses. However, everyday clinical practice easily shows that “bulky” tumor masses might have an entirely different impact on patients’ prognosis, depending on the metastatic site of involvement as in liver vs lung vs peritoneum vs others.

It is important to underline that the shorter follow-up time and the relatively low number of death events in 2021-2022 compared to the 2020 cohort might have affected OS results and comparisons.

Since the present study represents the joint effort of nationwide cooperation, it also accounts for regional variations in response to the COVID-19 pandemic, including geographic distribution and local governments’ crisis management. Interestingly, a higher reduction in terms of new CRC diagnoses between 2020 and 2019 was found in the regions of Northern Italy compared to Central Italy, with a rebound effect in the post-emergency pandemic phase. These findings should not surprise since Northern Italy was committed first by the COVID-19 pandemic in 2020.

We acknowledge that our work has potential limitations as a retrospective investigation. In the present study, patients with recurrent disease were excluded to analyze a homogeneous sample of new CRC diagnoses and to avoid potential biases related to the oncological management during the follow-up period for patients with previous CRC. This decision could be considered a potential limitation of the study since COVID-19 might have equally affected on diagnosis and treatment of CRC relapse. Nevertheless, as the cooperative effort of a multicentered national collaboration, our data provide valuable and thorough insight into cancer care across 3 years after the COVID-19 pandemic outbreak. Moreover, differently from informatics data analysis from National Cancer Registries, our real-world study, through the analysis of medical records of 1845 patients, is less affected by potential reporting biases during the frenetic times assessed.

Gathering together these findings, our study offers a valuable picture of the performance of Italian Oncology Departments to guarantee timely diagnosis, staging, and treatment for CRC patients across all the pandemic years. Despite this, with all the above-mentioned limitations, a certain independent negative prognostic effect in patients affected by mCRC managed during the pandemic years would seem to be pointed out. With this regard, it would be desirable in the future to learn from those virtuous experiences for which critical circumstances like the COVID-19 pandemic represented an opportunity to transform and renew screening services with a robust recovery plan and a clear practical implementation of a restart.^38^ This, together with the presence of properly and timely defined guidelines and recommendations for patient management during emergency situations, might help limit the risk of a negative prognostic impact.^15^

## Supplementary material

Supplementary material is available at *The Oncologist* online.

oyae310_suppl_Supplementary_Figures_1-6_Tables_1-2

## Data Availability

The data collected for this study could be available in a de-identified form after reasonable request.
