# Supplementary material for: Changes in clinical presentation, management, and survival outcomes in patients affected by colorectal cancer following COVID-19 pandemic
Source: Oncologist. 2024 Nov 26;30(7):oyae310. doi: 10.1093/oncolo/oyae310 (PMC12311278; doi:10.1093/oncolo/oyae310)
Supplement: oyae310_suppl_Supplementary_Figures_1-6_Tables_1-2 [file oyae310_suppl_supplementary_figures_1-6_tables_1-2.docx]

**Supplementary Material**


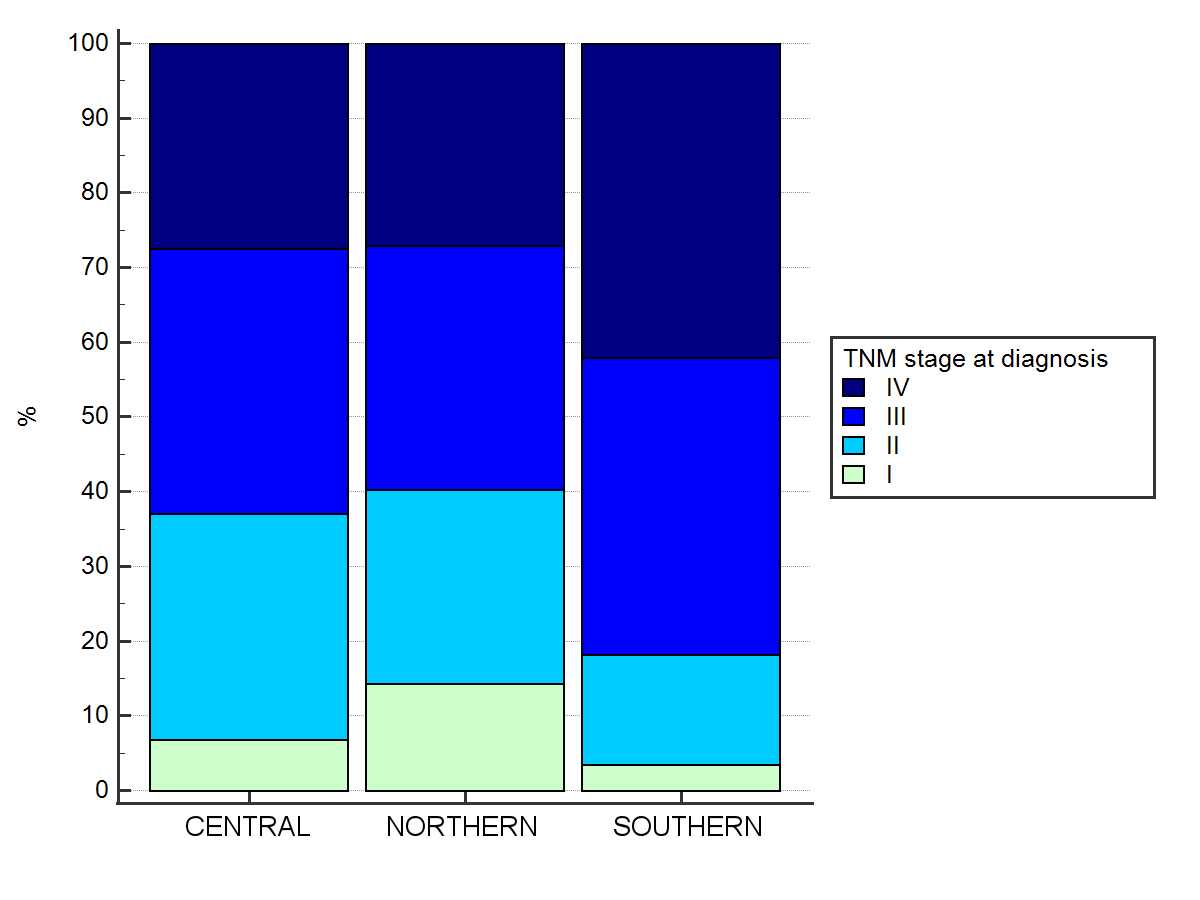


**Supplementary figure 1.** TNM stage at diagnosis according to Italian regions.


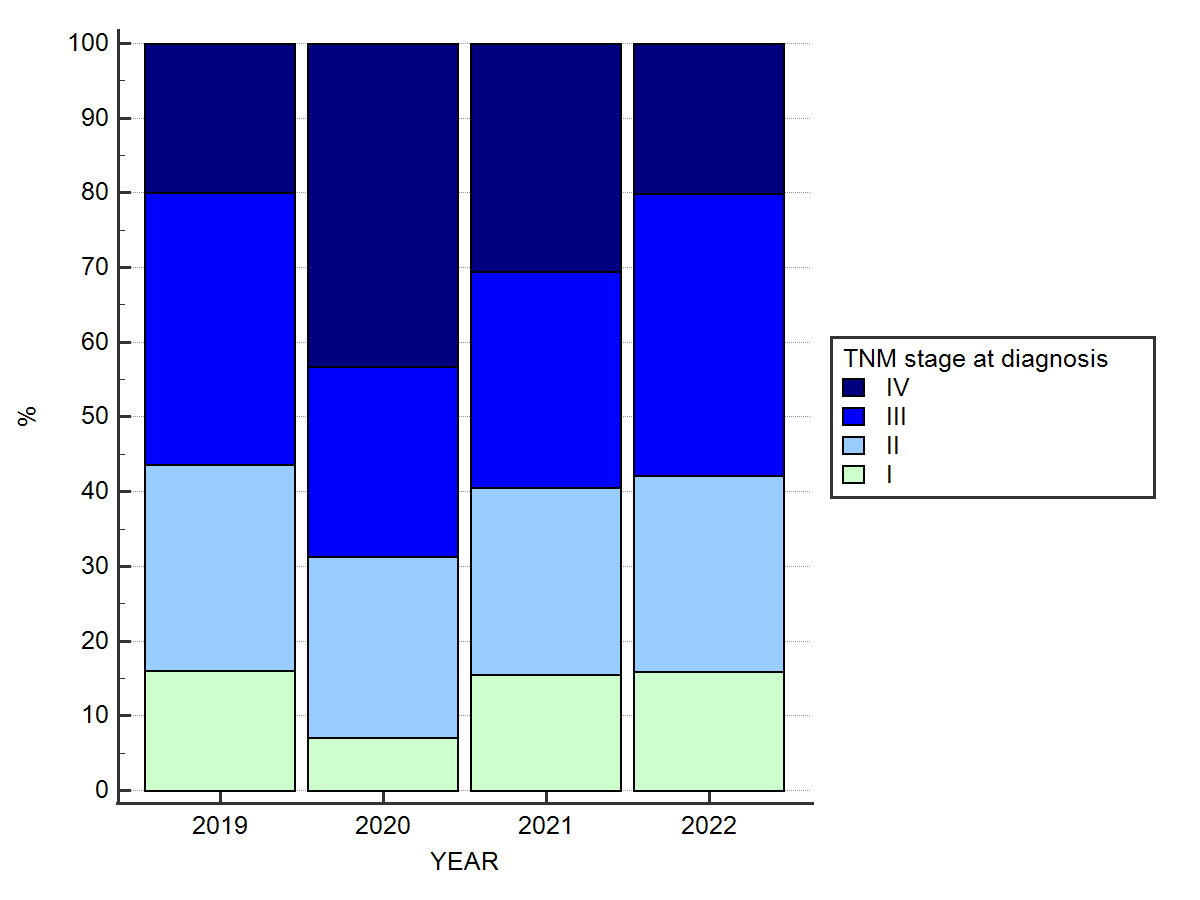


**Supplementary figure 2.** TNM stage at diagnosis according to year in Northern Italy


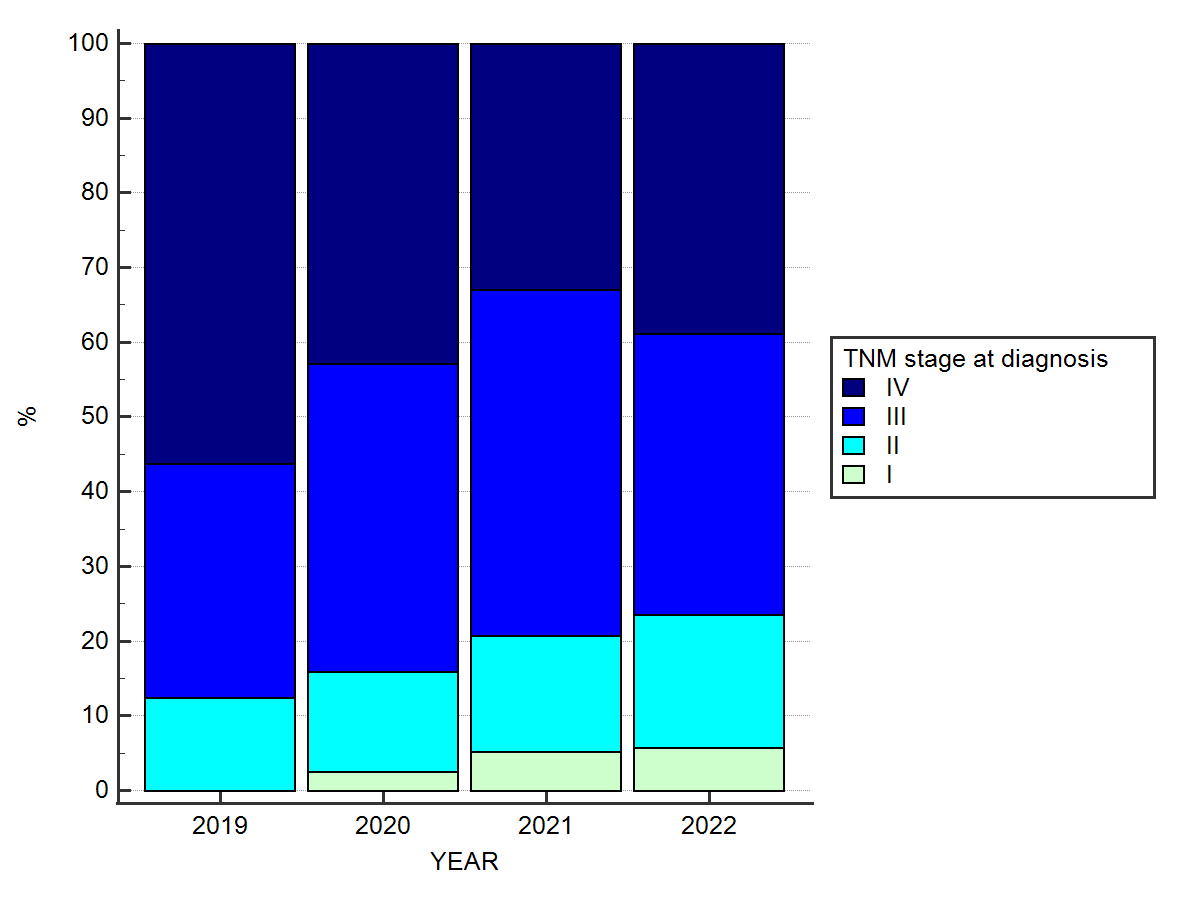


**Supplementary figure 3.** TNM stage at diagnosis according to year in Southern Italy

| **Time interval** | **2020**  **Median,**  **Days (IQR)** | **2021-2022**  **Median,**  **Days (IQR)** | **P value^a^** |
| --- | --- | --- | --- |
| Symptom onset/radiological diagnosis | 20 (58) | 23 (42) | *0.914* |
| Symptom onset/cytohistological diagnosis | 28 (62) | 26 (47) | *0.430* |
| Symptom onset/first oncological appointment | 69 (65) | 73 (63) | *0.323* |
| Cytohistological diagnosis/first oncological appointment | 30 (30) | 38 (33) | *<0.001* |
| Symptom onset/treatment start | 91 (78) | 94 (73) | *0.559* |
| Cytohistological diagnosis/treatment start | 49 (43) | 58 (38) | *<0.001* |
| First oncological appointment/treatment start | 14 (16) | 16 (18) | *0.007* |
| Treatment start/first radiological assessment | 96 (87) | 105 (81) | *0.027* |

**Supplementary table 1.** Temporal intervals between date of symptoms onset, radiological diagnosis, cytohistological diagnosis, first oncological appointment, treatment start, and first radiological reassessment between 2019-2020 and 2021-2022**.** IQR, interquartile range. ^a^Mann-Whitney U test comparing time intervals between 2020 and 2021-2022. P values were calculated excluding patients with unknown values. Statistically significant (P < 0.05).

| **Variable** | **Risk of death**  **(OS)** | | **Risk of disease progression/death**  **(PFS)** | |
| --- | --- | --- | --- | --- |
|  | Multivariable  Co-HR (95% CI) | P value | Multivariable  Co-HR (95% CI) | P value |
| **Region of Italy**  Central  Northern  Southern | 1  1.99 (1.31-3.0)  1.10 (0.75-1.6) | 0.001  0.614 | 1  1.43 (1.08-1.91)  1.32 (1.00-1.74) | 0.013  0.046 |
| **Year**  2019  2020  2021  2022 | 1  1.76 (1.12-2.8)  2.29 (1.37-3.8)  3.69 (1.83-7.4) | 0.014  0.002  <0.001 | 1  1.90 (1.36-2.66)  1.73 (1.23-2.42)  2.8 (1.82-4.30) | <0.001  0.002  <0.001 |
| **Molecular status**  RAS/BRAF wild-type  BRAF mutant  RAS mutant | 1  1.44 (0.67-3.1)  1.68 (1.20-2.40) | 0.345  0.003 | 1  2.38 (1.52-3.73)  1.56 (1.22-2.01) | <0.001  <0.001 |
| **MMR/MSI status**  pMMR/MSS  dMMR/MSI-h | 1  0.81 (0.32-2.0) | 0.644 | 1  0.50 (0.24-1.03) | 0.062 |
| **Sidedness**  Left  Right  Rectum | 1  0.80 (0.57-1.10)  0.98 (0.58-1.60) | 0.929  0.223 | 1  1.00 (0.77-1.29)  1.01 (0.69-1.47) | 0.966  0.972 |
| **ECOG-PS**  0  1  2-3 | 1  2.12 (1.52-2.9)  2.77 (1.28-6.0) | <0.001  0.009 | 1  1.56 (1.23-1.98)  1.73 (0.95-3.16) | <0.001  0.072 |
| **Age onset (years)**  50-75  >75  <50 | 1  1.09 (0.76-1.60)  0.82 (0.41-1.60) | 0.622  0.572 | 1  0.99 (0.74-1.32)  0.59 (0.37-0.94) | 0.954  0.026 |
| **Diagnosis performed during access to first aid**  No  Yes | 1  0.94 (0.67-1.3) | 0.747 | 1  1.11 (0.86-1.42) | 0.062 |
| **Gender**  Male  Female | 1  1.03 (0.75-1.4) | 0.857 | 1  1.03 (0.81-1.30) | 0.838 |

**Supplementary table 2.** Multivariable analysis for OS and PFS in the overall population.


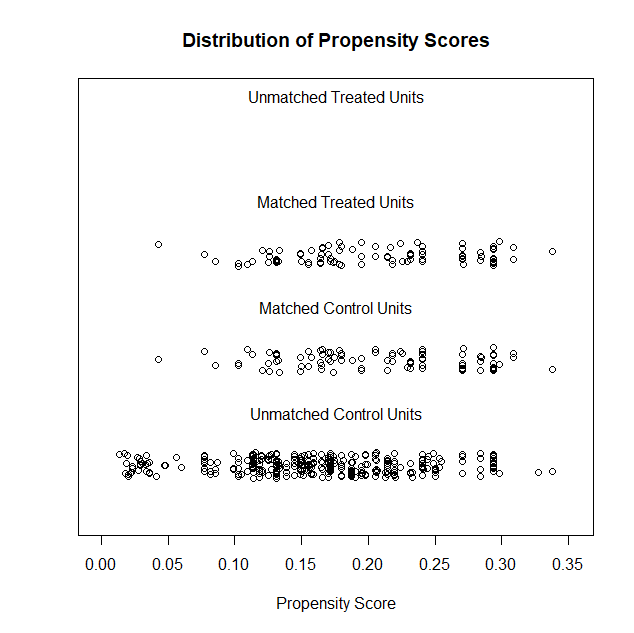


**Supplementary figure 4 A.** Jitter plot of the matched cohort.


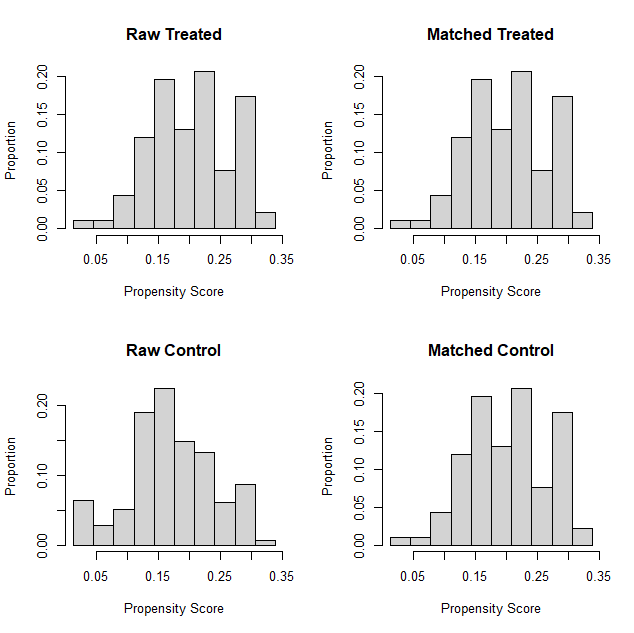


**Supplementary figure 4B.** Histogram plot of the matched cohort.


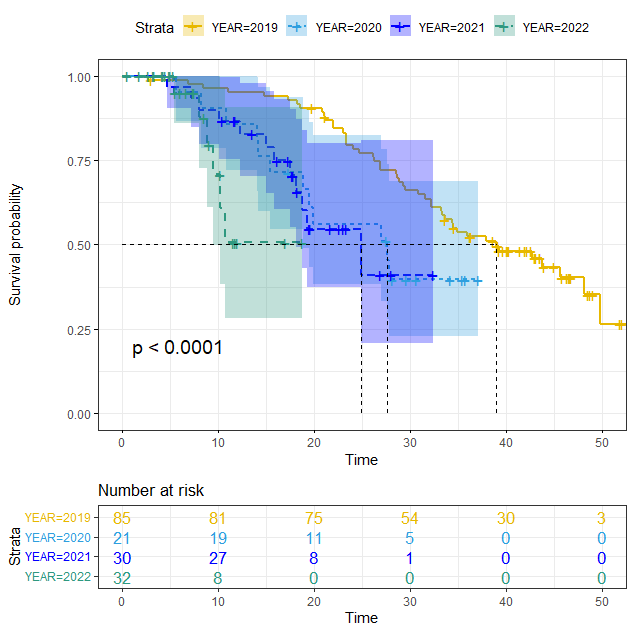


**Supplementary figure 5.** Kaplan-Meir curves for OS in the matched cohort.


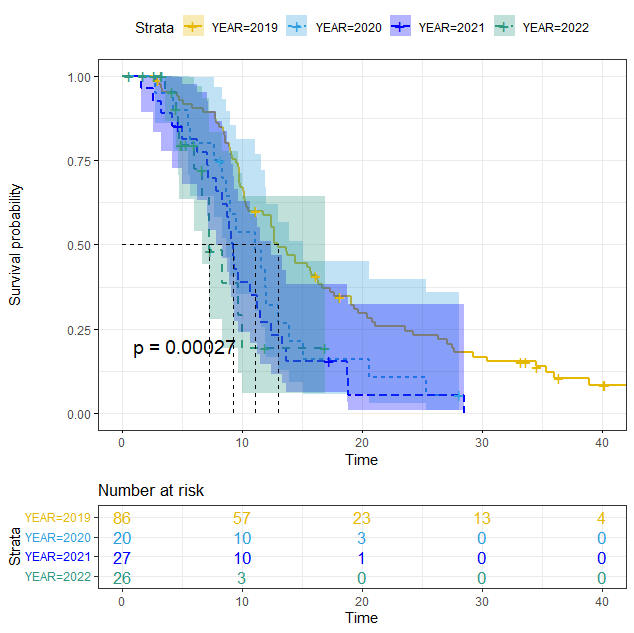


**Supplementary figure 6.** Kaplan-Meir curves for PFS in the matched cohort.
